# Human adaptation to the hypoxia of high altitude: the Tibetan paradigm from the pregenomic to the postgenomic era

**DOI:** 10.1152/japplphysiol.00605.2013

**Published:** 2013-11-07

**Authors:** Nayia Petousi, Peter A. Robbins

**Affiliations:** Department of Physiology, Anatomy and Genetics, University of Oxford, Oxford, United Kingdom

**Keywords:** hypoxia-inducible factor, *EPAS1*, *EGLN1*, evolution, Tibetan, adaptation

## Abstract

The Tibetan Plateau is one of the highest regions on Earth. Tibetan highlanders are adapted to life and reproduction in a hypoxic environment and possess a suite of distinctive physiological traits. Recent studies have identified genomic loci that have undergone natural selection in Tibetans. Two of these loci, *EGLN1* and *EPAS1*, encode major components of the hypoxia-inducible factor transcriptional system, which has a central role in oxygen sensing and coordinating an organism's response to hypoxia, as evidenced by studies in humans and mice. An association between genetic variants within these genes and hemoglobin concentration in Tibetans at high altitude was demonstrated in some of the studies (8, 80, 96). Nevertheless, the functional variants within these genes and the underlying mechanisms of action are still not known. Furthermore, there are a number of other possible phenotypic traits, besides hemoglobin concentration, upon which natural selection may have acted. Integration of studies at the genomic level with functional molecular studies and studies in systems physiology has the potential to provide further understanding of human evolution in response to high-altitude hypoxia. The Tibetan paradigm provides further insight on the role of the hypoxia-inducible factor system in humans in relation to oxygen homeostasis.

the severely reduced oxygen availability at high altitude, termed hypobaric hypoxia, presents a significant challenge to the ability of humans residing there to live and reproduce. As such, it is likely to have acted as an agent of natural selection. Three human populations have lived at high altitude for millennia: Andeans on the Andean Altiplano, Tibetans on the Tibetan Plateau, and Ethiopians on the Semian Plateau. Of these, Tibetans appear to have lived at high altitude the longest. According to archeological and other data, e.g., genetic studies, humans have lived on the Tibetan Plateau, one of the highest regions on Earth with an average elevation of ∼4,000 m, a barometric pressure of <500 mmHg, and an inspired partial pressure of oxygen of ∼80 Torr, for at least 25,000 yr (1, 2, 71, 101). In contrast, it is estimated that the Andean Altiplano was first populated approximately only 11,000 yr ago (2), while the Amhara population in Ethiopia is assumed to have settled at high altitude for ∼5,000 yr (3). This suggests that Tibetans will have had more time and opportunity for natural selection in response to a hypoxic environment than any other high-altitude human population.

Over the last few decades, much research has been done to investigate how Tibetans are adapted to life in a hypoxic environment. Compared with other high-altitude populations and lowland natives who emigrated to high altitude, Tibetans were found to exhibit distinctive physiological traits and to be resistant to certain pathophysiological processes. While in a number of studies these traits exhibited a great degree of heritability (7, 11, 12, 20), it was largely unknown whether the distinctive high-altitude Tibetan phenotype was the result of natural selection. It is only recently, within the last few years, i.e., in the postgenomic era, that advances in genomic technology and science have made it possible to gather evidence of natural selection in Tibetans and to implicate certain genes in their genetic adaptation. Nevertheless, the functional variants within these genes and the underlying mechanisms involved are still unknown.

In this review, we highlight the dominant physiological features of Tibetans living at high altitude. We then review the findings of recent studies investigating genetic selection in Tibetans. We discuss how the genes identified as strong candidates for a role in Tibetans' evolutionary adaptation to high altitude are involved in human physiological processes related to oxygen homeostasis. We discuss and speculate on the potential physiological trait(s) upon which natural selection has acted. Finally, we consider how the evolutionary paradigm of the Tibetan high-altitude adaptation provides insights and raises questions on the role of the hypoxia-inducible factor (HIF) system in humans who live at sea level.

## TIBETAN PHYSIOLOGY AT HIGH ALTITUDE

The human response to hypoxia is characterized by systemic changes in cardiovascular, respiratory, and hematopoietic physiology, which affect convective oxygen transport. Central to the Tibetan high-altitude phenotype is the fact that certain components of convective oxygen transport are markedly different in Tibetans compared with other humans living at high altitude. In this review, most comparisons are made between Tibetans and Andeans, as the physiology of these two populations has been extensively studied. In contrast, significantly less is known about the physiology of the Ethiopian highlanders.

An immediate rise in ventilation is the most obvious response of human lowlanders exposed to acute high-altitude hypoxia, followed by more complex time-dependent changes with prolonged exposure (69). Tibetan highlanders resemble acclimatized newcomers in that they maintain a high resting ventilation and brisk hypoxic ventilatory sensitivities (32, 36), whereas Andeans exhibit a considerable degree of ventilatory blunting (46, 78). Indeed, Tibetan highlanders had elevated resting ventilation and augmented acute hypoxic ventilatory responses when compared directly with Han Chinese long-term residents of the same altitude (102) or Bolivian Aymaras resident in the Andes (11).

Hypoxia constricts rather than dilates the human pulmonary vasculature (59). Regional hypoxic pulmonary vasoconstriction (HPV) distributes blood away from hypoxic regions of the lung and can be beneficial in maintaining perfusion-ventilation matching. However, a consequence of HPV is that global hypoxia, such as that experienced at high altitude, leads to pulmonary arterial hypertension and right heart strain. Tibetan highlanders, in contrast to their Andean counterparts or other high-altitude residents, exhibit a relative resistance to developing pulmonary hypertension. This was demonstrated directly in a study of five Tibetans resident at 3,658 m who had pulmonary arterial pressures within sea level norms that were little changed by additional hypoxia (27). Consistent with this is the demonstration of elevated nitric oxide, a vasodilator, in the lungs of Tibetans compared with Andean highlanders and lowlanders at sea level (10, 34). Furthermore, a study demonstrated lack of smooth muscle in the small pulmonary arteries in Tibetan men at 3,600 m, consistent with the absence of pronounced pulmonary vascular remodeling and hypertrophy that accompanies pulmonary hypertension (30).

**Fig. 1.**
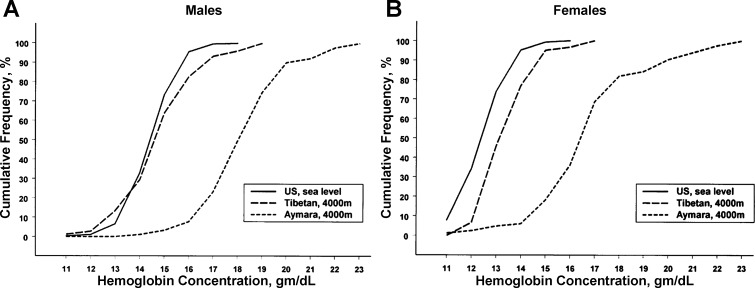
Cumulative frequency distribution of hemoglobin concentrations in US residents at sea level and Tibetan and Bolivian Aymara natives at 4,000 m. *A*: results for men. *B*: results for women. [From Beall et al. (7).]

A hallmark of high-altitude hypoxic exposure in humans is the rise in red cell mass accompanied by elevated hemoglobin (Hb) concentration and hematocrit, which is associated with a rise in blood erythropoietin (Epo) levels (73). Perhaps the most striking phenotypic difference between Tibetan highlanders and other high-altitude residents is their blunted erythropoietic response to hypoxia. This is evident by the fact that, at similar high altitudes, Tibetans have significantly lower Hb concentrations, typically 1–3.5 g/dl lower, than their Andean counterparts or Han Chinese migrants to high altitude (7, 26, 55, 90). Tibetans exhibit little or no increase in Hb concentration with increasing altitude (6, 91), and, at 4,000 m, both male and female Tibetans have Hb concentrations comparable to those of US sea level residents (7) (see Fig. 1).

Chronic mountain sickness (CMS) is a high-altitude disorder linked with the above components of oxygen transport. It is a syndrome of adaptive failure to chronic hypoxia characterized by excessive erythrocytosis with abnormally high Hb and hematocrit levels, hypoventilation, pulmonary hypertension, and eventually right heart failure (47, 53). It occurs in adults after prolonged residence at high altitude and is associated with significant morbidity and mortality (40, 60). Of interest is the remarkably low prevalence of CMS in Tibetan highlanders compared with other high-altitude populations, such as Andeans or Han Chinese migrants to high altitude (54). The overall prevalence of CMS in Tibetans living on the Qinghai-Tibetan plateau was 1.2% compared with 5.6% in Han Chinese (92). Using the same criteria as Monge et al. (53) to define CMS, i.e., [Hb] >21.3 g/dl and arterial O_2_ saturation < 83%, a lower prevalence was reported in Tibetans (0.9%) at 4,300 m (Madu) (93) compared with Peruvian Quechuas (15.6%) at the same altitude (Cerro De Pasco) (53).

**Fig. 2.**
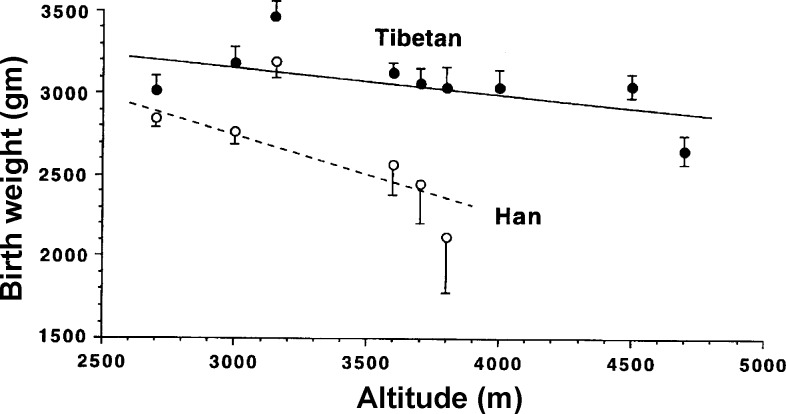
Birth weight plotted against altitude of residence for Tibetans and Han Chinese, showing a significantly smaller birth weight fall with increasing altitude in Tibetans than in Han Chinese. Values are means ± SE. [From Moore et al. (57).]

Another characteristic of the Tibetan population is a relative protection against the occurrence of intrauterine growth restriction (IUGR), which is associated with low birth weight at high altitude. It is well recognized that reproductive success is more difficult at high than low altitude, especially among nonnatives (43). Birth weight progressively reduces with increasing altitude across populations (41, 56, 97). However, the magnitude of this fall in birth weight varies among populations: it is smaller in populations that have lived for multiple generations at high altitude (31, 57, 99), such as Tibetans and Andeans, suggesting that genetic factors play a role. Not only were heavier birth weights observed in Tibetans compared with Han Chinese at the same altitude, but the pre- and postnatal mortality was threefold higher in Han Chinese than Tibetans (57). Figure 2 shows the birth weight reduction with altitude for Tibetan and Han Chinese. To explain these differences, various studies have focused on oxygen delivery to the placenta and found that they are associated with differences in uterine artery (UA) blood flow, as opposed to changes in ventilation, Hb concentration, or Hb saturation in pregnant women. Higher UA flow velocities (58) and larger UA diameters (17) were observed in pregnant Tibetan compared with Han Chinese women. Analogous results have been found in studies of Andean populations; a doubling of the UA diameter during pregnancy was demonstrated in Andean women but not in European women at high altitude, an effect seen only under circumstances of chronic hypoxia but not at low altitude (42, 89, 100). The underlying mechanisms for these observations, and whether they are the same in Tibetans and Andeans, are not currently known.

The phenotypic characteristics of Ethiopian highlanders have not been examined as extensively. Amhara Ethiopians at 3,500 m were reported to have low Hb concentrations comparable with those of lowlanders (3, 9), resembling Tibetans; however, Scheinfeldt et al. (75) observed higher Hb concentration in high-altitude Amharas compared with lowlanders. Pulmonary arterial pressure, on the other hand, was found to be elevated in Ahmara highlanders (35).

The distinctive oxygen-transport traits found in Tibetans compared with other high-altitude populations, together with reports of intrapopulation heritability, have been interpreted as demonstrating the presence of genetic influences on high-altitude adaptation. Moreover, the association of these traits to disorders such as CMS and IUGR, which have the potential to influence reproduction and survival, suggests that some of these phenotypic characteristics, alone or in combination, may be offering an evolutionary survival benefit to Tibetans.

## STUDIES OF THE TIBETAN GENOME: EVIDENCE OF NATURAL SELECTION

Recent genome-wide analyses in Tibetans provided the first lines of evidence that this population has undergone genetic adaptation to high altitude. These studies used different methodologies to compare the Tibetan genome with genomes of closely related ethnic backgrounds, such as the Han Chinese (8, 13, 62, 80, 88, 95, 96). The rationale was that any differences captured in the genetic structure between the two populations are likely to have arisen through natural selection in response to the hypoxic environment inhabited by the Tibetans. These studies have been extensively reviewed (5, 51, 76, 79). The studies are summarized in Table 1, and the key findings are discussed below.

**Table 1.** Summary of genome-wide studies of high altitude adaptation in Tibetans

Study	Populations	Methodology (Approach and Platform)	HIF Pathway Genes Identified
Simonson et al. (80)	1. Tibetan highlanders from Qinghai Province (*n* = 31) vs. lowland HapMap CHB and JPT (*n* = 90)	1. XP-EHH, iHS (Affymetrix 6.0 SNP array)	*EGLN1 (& EPAS1)*
	2. Tibetan highlanders (*n* = 29) from Qinghai Province	2. Candidate gene association study (Hb concentration)	
Beall et al. (8)	1. Tibetan highlanders from Yunnan Province (*n* = 35) vs. lowland HapMap CHB (*n* = 84)	1. GWADS (Illumina 610-Quad SNP array)	*EPAS1*
	2. Tibetan highlanders from Mag Xiang, Tibet Autonomous Region (*n* = 70) and from Zhaxizong Xiang, Tibet Autonomous Region (*n* = 91)	2. Candidate gene association study (Hb concentration)	
Yi et al. (96)	1. Tibetan highlanders from Tibetan Autonomous Region (*n* = 50) vs. lowland HapMap CHB (*n* = 40) and Danish controls (*n* = 100)	1. PBS (Exome Sequencing, Illumina)	*EPAS1*
	2. Tibetan highlanders from Tibetan Autonomous Region (*n* = 366)	2. Candidate gene association study (erythrocyte count)	
Bigham et al. (13)	Tibetan highlanders from Tibetan Autonomous region (*n* = 50) vs. lowland HapMap East Asian (*n* = 60) and European controls (*n* = 90)	LSBL, lnRH, Taj D, WGLRH (Affymetrix 6.0 SNP array)	*EGLN1 (& EPAS1)*
Peng et al. (62)	1. Tibetan highlanders from Qinghai Province (*n* = 50) vs. lowland HapMap CHB/JPT	1. XP-CLR, F_ST_ (Affymetrix 6.0 SNP array)	*EPAS1 (& EGLN1)*
	2. Tibetan highlanders from Qinghai Province (*n* = 50) vs. lowland HapMap CHB/JPT/YRI/CEU	2. Full-length sequencing of *EPAS1* (haplotype construction, F_ST_)	
	3. Tibetan highlanders from Tibetan Autonomous Region, Qinghai, and Yunnan Provinces (*n* = 1,334)	3. Candidate gene SNP genotyping for allele frequency comparisons	
Xu et al. (95)	Tibetan highlanders from Tibet (Shannan, Rikaza, Linzhi, Lasha, and Changdu) (*n* = 46) vs. Han Chinese (*n* = 92), YRI (*n* = 60), CEU (*n* = 60), and JPT (*n* = 44) from HapMap	F_ST_, iHS, XP-EHH (Affymetrix 6.0 SNP array), haplotype construction	*EPAS1& EGLN1*
Wang et al. (88)	Tibetan highlanders from near Lhasa (*n* = 30) vs. lowland HapMap CHB (*n* = 45), JPT (*n* = 45), CEU (*n* = 59), and YRI (*n* = 60) and East Asian from HGDP	F_ST_, iHS, XP-EHH (Illumina 1M)	*EPAS1 (& EGLN1)*

HIF, hypoxia-inducible factor; CHB, Han Chinese in Beijing, China; JPT, Japanese in Tokyo, Japan; YRI, Yoruba in Ibadan, Nigeria; CEU, Utah residents with Northern and Western European ancestry; HGDP, human genome diversity project; XP-EHH, cross-population extended haplotype homozygosity; iHS, integrated haplotype score; GWADS, genome-wide allelic differentiation scan; PBS, population branch statistic; LSBL, locus-specific branch length; lnRH, natural logarithm of ratio of heterozygosities; Taj D, Tajima's D statistic; WGLRH, whole genome long-range haplotype; XP-CLR, cross-population composite likelihood ratio test; F_ST_, fixation index. The basis of these and other techniques are described in recent reviews (15, 80).

Simonson et al. (80) performed a genome-wide comparison of Tibetan and Han Chinese or Japanese populations. They identified chromosomal regions of positive selective sweeps by calculating two separate genomic statistics: the cross-population extended haplotype homozygosity statistic (XP-EHH), and the integrated haplotype score (iHS) for 200-kb non-overlapping genomic regions. The basis of these and other techniques is described in recent reviews (15, 82). They then scanned the positive regions for 247 a priori selected candidate genes (based on their involvement in oxygen homeostasis) and found 10 genes that were located in or near these regions. Among these genes were *EPAS1*, *EGLN1*, and *PPARA*, with *EGLN1* identified by both genomic statistics. In addition, in a cohort of 29 Tibetans, genetic variants in the latter two genes were associated with Hb concentration such that the selected haplotype in each gene was correlated with low Hb concentration. However, people with excessive erythrocytosis or anemia were not excluded from this genotype-phenotype analysis.

**Fig. 3.**
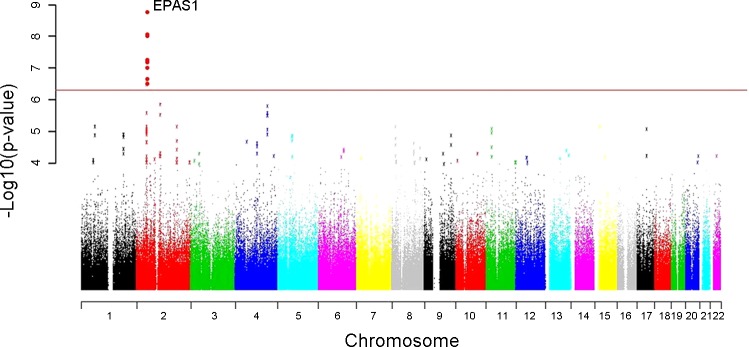
Genome-wide allelic differentiation scans comparing Tibetan highlanders and HapMap Han Chinese. Eight single nucleotide polymorphisms near *EPAS1* achieved genome-wide significance. [From Beall et al. (8).]

Beall et al. (8) used a genome-wide allelic differentiation scan (GWADS) to compare ∼500,000 single nucleotide polymorphisms (SNPs) between Tibetan high-altitude natives (from the Yunnan province) and lowland Han Chinese (see Fig. 3). This provided a completely unbiased genome-wide analysis investigating genetic selection, with no a priori assumptions or hypotheses. They found eight SNPs that achieved genome-wide significance (*P* values ranging from 2.81 × 10^−7^ to 1.49 × 10^−9^) in terms of their divergence between the two ethnic groups, which clustered within ∼235 kb on chromosome 2, just downstream of *EPAS1*. These SNPs were found to be in high linkage disequilibrium (LD) with one another: i.e., they were associated with each other in a nonrandom way such that they occurred together more often than expected by chance. This extended haplotype was present at high frequencies in Tibetans (46%) and low frequencies (2%) in Han Chinese. In addition, through candidate gene approaches in two further separate cohorts of Tibetans, they identified ∼30 SNPs in *EPAS1* in high LD (consistent with a dominant variant hypothesis) that correlated significantly with Hb concentration. The correlation was such that major allele (alias “Tibetan” allele) homozygotes averaged a Hb concentration of ∼1 g/dl lower than the heterozygotes in each separate cohort. These *EPAS1* SNPs were found at higher frequencies in Tibetans than in Han Chinese and were in high LD with the signal from the genome-wide allelic differentiation scan, thus providing evidence for selection on *EPAS1* associated with low Hb concentration in Tibetan highlanders.

Yi et al. (96) sequenced 50 Tibetan exomes and compared the sequencing data with Han Chinese and Danish populations to identify changes in allelic frequencies consistent with genetic adaptation in Tibetans. Using population branch statistics (PBS), they identified *EPAS1* as the strongest candidate gene for natural selection. In addition, the most differentiated (in terms of allelic frequency) *EPAS1* variant was correlated with erythrocyte count within a larger Tibetan cohort. This was an intronic *EPAS1* SNP, which was captured by this exome-targeted approach and was found at 87% frequency in Tibetans compared with 9% in Han Chinese. Interestingly, no coding genetic variants were identified to be highly differentiated between the populations. This leads to suggest that adaptation to high altitude has not proceeded by way of selection on coding variants that might be expected to alter protein structure and function. Based on their analyses, they estimated a Tibetan-Han divergence time of 2,750 yr, which is far more recent than previously estimated based on archeological data or other analyses (1, 62, 101). This short divergence time has been disputed; it has been argued that it may be related to a complex mosaic of the Tibetan population created by multiple migrations at different times into the Plateau and to the small sample sizes in the study (1).

Xu et al. (95) compared genome-wide allelic frequencies between Tibetan and Han Chinese and identified 6 *EGLN1* SNPs and 25 *EPAS1* SNPs among the top differentiated SNPs (top 0.0001% SNPs with F_ST_ statistic > 0.3). They went on to identify positive selective sweeps and dominant haplotypes in *EPAS1* and *EGLN1* genes in Tibetans and also showed a correlation of the prevalence of the dominant haplotypes for these genes with altitude of residence.

Peng et al. (62) SNP-typed 50 Tibetan individuals and compared the results with data from HapMap Han Chinese, identifying *EPAS1* as one of the genes that underwent a selective sweep in Tibetans. They also sequenced the full length of the *EPAS1* gene in these individuals and showed marked allelic differentiation at this locus between Tibetans and Han Chinese. They constructed haplotypes, and further analysis allowed an estimation of a divergence time for Tibetans that was sixfold greater than that of Yi et al. (96) and consistent with previous archeological and genetic reports (1, 2, 101). They also genotyped three SNPs from each of *EPAS1*, *EGLN1*, and *PPARA* in a large cohort of 1,334 Tibetans. They found significant differences in allele frequencies for the *EPAS1* SNPs between Tibetan and Han Chinese/Japanese and also for one of the SNPs in *EGLN1*.

Wang et al. (88) performed a number of tests of selection using genome-wide SNP data from 30 Tibetans living near Lhasa and identified the *EPAS1* locus as the strongest signal of positive selection in the Tibetan genome. The second most significant genomic region with evidence of positive selection was a region containing the *EGLN1* gene.

Bigham et al. (13) performed high-density genome scans and applied four population genetic statistics to identify selection-nominated candidate genes and candidate regions for two high-altitude populations, Andeans and Tibetans. These two populations were studied separately. They found different patterns of positive selection for the two populations. In Tibetans, a genomic region containing *EPAS1* exhibited significant variation between Tibetans and the lowland comparators, HapMap Asians. Interestingly, *EGLN1* showed evidence of positive selection in both Tibetans and Andeans, although the SNP frequencies and haplotypes are different between the two populations.

While *EGLN1* was identified as undergoing selection in both Tibetans and Andeans, neither *EPAS1* or *EGLN1* featured as candidates in recent genomic studies in Ethiopian highlanders (3, 37, 75). Other, different, candidate genes have been found in these studies. *PPARA*, however, which was previously reported as a selection candidate in Tibetans (80), was also identified as a selection candidate gene in highland Ethiopians with a marginal association with Hb concentration (75). Thus different high-altitude populations evolved independently to adapt to the challenges of a hypoxic environment. However, of interest is that, in the study by Huerta-Sanchez et al. (37), the top candidate gene, *DEC1*, is functionally related to the oxygen-sensing pathway; it is transcriptionally regulated by HIF-1α, directly binds HIF-1α, downregulates HIF-1α and HIF-2α protein expression, and represses many HIF-target genes. It is likely these interpopulation differences in genetic adaptation relate to the length of high-altitude inhabitation, the severity of the hypoxic stimulus, the different genetic backgrounds, different opportunities for admixture with lowlanders, and evolutionary chance.

Additional genes that may have undergone selection in Tibetans with rather weaker signals of selection do exist and have been reported in the various studies (29, 80, 96). However, what is striking when considering the convergent findings of all of the studies to date is the independent identification of positive selection in Tibetans (from different geographic locations) at genomic loci that involve *EPAS1* (which codes for HIF-2α) and *EGLN1* [which codes for prolyl hydroxylase domain (PHD) 2], both key components of the HIF pathway, thus implicating these genes in the genetic adaptation of Tibetans to high altitude.

## EPAS1 AND EGLN1: THE HIF TRANSCRIPTIONAL PATHWAY

HIFs are a family of transcription factors that coordinate oxygen sensing and intracellular responses to hypoxia through regulation of expression of hundreds of genes belonging to biological pathways such as energy metabolism, angiogenesis, erythropoiesis, iron homeostasis, apoptosis, and others (77).

**Fig. 4.**
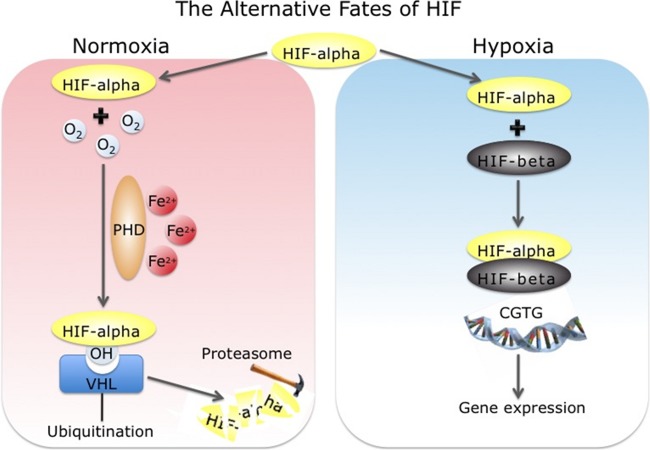
Schematic representation of the regulation of hypoxia-inducible factor (HIF)-α by hypoxia. In the presence of oxygen (O_2_) and iron (Fe^2+^), prolyl hydroxylase domain (PHD) enzymes hydroxylate specific proline residues in HIF-α, increasing the binding of the Von Hippel-Lindau (VHL) tumor suppressor protein. This targets HIF-α for ubiquitination and mediates its proteosmal degradation. In hypoxia, HIF-α accumulates, dimerizes with HIF-β, binds to DNA, and transcriptionally regulates hypoxia-responsive genes. [Courtesy of Dr. Federico Formenti, University of Oxford, Oxford, UK.]

*EPAS1* encodes HIF-2α (87), which is one of three HIF-α subunit isoforms. In the presence of oxygen, HIF-α is hydroxylated at two proline residues by PHD enzymes, of which there are three isoforms, PHD1, PHD2 (coded by the *EGLN1* gene), and PHD3, in an oxygen-dependent manner (22, 38, 39). This hydroxylation enhances the binding of the von Hippel-Lindau (VHL) protein to HIF-α, which in turn enhances HIF-α ubiquitination, resulting in its subsequent rapid proteasomal degradation (18, 39). In hypoxia, the reduced hydroxylase activity of the PHD enzymes results in the stabilization and accumulation of the HIF-α subunits, which then heterodimerize with HIF-β and transcriptionally activate target genes (Fig. 4). Evidence for a central role of the HIF pathway, and more specifically for *EPAS1* (HIF-2α) and *EGLN1* (PHD2), in systemic erythropoiesis, but also in other aspects of integrative physiology related to oxygen homeostasis, comes from studying patients with genetic abnormalities in the HIF system and from genetic mouse models. The most extensively studied patients are those with Chuvash polycythemia, who are homozygous for a hypomorphic allele of the VHL tumor suppressor, which impairs HIF-1α and HIF-2α degradation (4). These patients have excessive erythrocytosis with high Hb and hematocrit levels due to an upregulation of Epo production. Apart from *VHL*, gain-of-function mutations in *EPAS1* leading to stabilization of HIF-2α (64, 65, 67) and mutations in *EGLN1* associated with diminished activity of PHD2 (45, 66) have also been identified in patients with excessive erythrocytosis.

Further phenotyping has shown that patients with Chuvash polycythemia have abnormal cardiopulmonary physiology. They have pulmonary hypertension, increased resting ventilation, and, upon exposure to acute systemic hypoxia, they exhibit an exaggerated pulmonary vascular response and an exaggerated ventilatory sensitivity (81). In addition, they have abnormal metabolism during exercise with a reduced maximal exercise capacity, an increased exercise-induced lactate accumulation, and an early and marked phosphocreatine depletion and acidosis in skeletal muscle (24). In contrast, patients with gain-of-function HIF-2α mutations have a more restricted cardiopulmonary phenotype of pulmonary hypertension and exaggerated pulmonary vascular responses to acute hypoxia (23, 25).

Studies in genetically modified mice further elucidated the role of the HIF pathway in systemic physiology. Both HIF-1α- and HIF-2α-null mice die during embryogenesis (61, 74). Postnatal deletion of HIF-2α in a conditional mouse model resulted in anemia and demonstrated that HIF-2α, rather than HIF-1α, is the critical isoform regulating erythropoiesis in adults (28). Mice with heterozygous deficiency for functional HIF genes, either HIF-1α (98) or HIF-2α (16), developed less polycythemia and pulmonary hypertension in response to hypoxia. Mouse models of Chuvash polycythemia confirmed the phenotype of increased ventilation and pulmonary hypertension found in humans and further demonstrated that the effects seen in the disease are primarily HIF-2α driven as opposed to HIF-1α driven (33). Similarly, a mouse model of the human HIF-2α gain-of-function mutation recapitulated the phenotype of excessive erythrocytosis and pulmonary hypertension in a dose-dependent manner (86). Knockout mice with heterozygous loss of HIF-1α had depressed carotid body oxygen sensitivity and abnormal ventilatory acclimatization to chronic hypoxia (44). In contrast, HIF-2α^+/−^ mice manifested heightened carotid responses to hypoxia, highlighting potentially different roles of HIF-1 and HIF-2 in systemic physiology (63). Germline PHD2^−/−^ mice are not viable (85). PHD2 conditional knockout mice developed excessive erythrocytosis and increased vascular growth (83, 84), and mice heterozygous for PHD2 had increased ventilatory sensitivity to hypoxia and carotid body hyperplasia (14).

The above findings highlight the key role of the HIF pathway as a master controller of oxygen sensing and regulator of systemic physiology in humans and mice. It is evident that perturbations of the HIF pathway, whether leading to an upregulation or a downregulation of the hypoxic HIF response, act on components of convective oxygen transport such as ventilation, hypoxic pulmonary vascular response, and erythropoiesis and produce phenotypic changes. Interestingly, it is these components of oxygen transport that are distinctive in Tibetan highlanders and make up their high-altitude phenotype, as previously mentioned, alluding to a role of the HIF pathway in their adaptation to hypoxia. This is discussed below, where we consider the potential physiological trait(s) upon which natural selection may have acted.

## CONCLUSIONS AND FUTURE CONSIDERATIONS

We have briefly reviewed the evidence that genetic variation in the HIF pathway affects hematopoietic and cardiopulmonary physiology in humans. The similarities between the phenotype of lowland patients with genetic mutations in the HIF pathway that “upregulate” the HIF response with the phenotype of patients with CMS at high altitude are remarkable. Coupling these observations with the recent studies identifying *EPAS1* and *EGLN1* as loci that have undergone recent positive selection in Tibetans (8, 13, 62, 80, 95, 96) suggests that natural selection to hypoxia in Tibetans may have occurred through genetic variation in the HIF pathway, which operated on related integrative-physiology phenotypes. Several questions remain unanswered.

One question relates to the identification of the “substrate” of natural selection, in other words, the phenotype on which selection occurred. In some reports, the putatively selected “Tibetan” genetic variants were associated with lower Hb concentrations at high altitude (8, 80, 96). Several possibilities exist. One is that blunted erythropoiesis may be beneficial for highlanders, and that low Hb concentration may be the “selected” phenotype. Arguments in favor of this are the association of excessive erythrocytosis with CMS, which poses significant morbidity and mortality risks, and the remarkably low prevalence of CMS in the Tibetan population. On the other hand, the higher Hb and hematocrit levels found in the Andean populations are not always associated with disease and do not preclude the Andeans from being a growing population at high altitude, suggesting that blunted erythropoiesis alone may not be a sufficient enough advantage to drive genetic selection in Tibetans. Another possibility is that the phenotype of low-Hb concentration is selected only as part of a more complex integrative phenotype that offers a survival advantage at high altitude, owing to the pleiotropic effects of the HIF pathway, and of *EPAS1* in particular. It is even possible that the hematopoietic phenotype may actually be a secondary outcome, or even a “side effect”, of the selection process, which may have acted on some other aspect of the phenotype. Apart from affecting cardiopulmonary physiology, *EPAS1* plays roles in placenta and embryonic development (19, 52, 70) and may be involved in IUGR (21). Of particular interest is that the placenta is one of the tissues where high expression of HIF-2α is found (72). The increased reproductive ability of Tibetans, as evident by the low pre- and postnatal mortality compared with Han Chinese high-altitude residents, coupled with their resistance to IUGR, suggests that natural selection on *EPAS1* may have also operated via effects during pregnancy on fetal growth. The study of associations between birth weight at high altitude and genotype is a potentially fruitful area of investigation.

A further question to consider is whether the effects of the genetic variation in *EPAS1* and *EGLN1* selected for in Tibetans are functional and operate in generating a phenotype only in conditions of severe and ongoing, i.e., for years, environmental hypoxia. It is of interest that, while genetic variants in *EPAS1* and *EGLN1* were significantly correlated with Hb concentration in Tibetan highlanders, neither *EPAS1* nor *EGLN1* featured in any of a number of genome-wide studies exploring genetic determinants of Hb concentration at sea level (22–24). This suggests that either the genetic variation in *EPAS1* or *EGLN1* relates to a hematopoietic phenotype only in conditions of severe and prolonged environmental hypoxia, or that the “functional” genetic variants in these genes are unique to Tibetans, naturally selected by living for generations in hypoxia. This raises an interesting question as to whether lessons can be learned from the Tibetan paradigm on the role of the HIF system near sea level. An ensuing fundamental question is to what degree the physiological responses that lowland humans exhibit when exposed to high altitude, e.g., erythropoietic and cardiopulmonary, can be considered adaptive to a reduction in ambient oxygen as opposed to “mishaps” arising from oxygen-sensing mechanisms that have been optimized to operate at “normal” oxygen levels found at sea level. Accordingly, the HIF system in humans may have been optimized to sculpt structure and physiological function using oxygen as a guiding signal rather than to respond to environmental hypoxic challenges. For example, reduced oxygen delivery to the kidneys is used as a guiding signal to stimulate Epo production through an upregulated HIF system to restore reductions in Hb or hematocrit levels, e.g., in anemia or hemorrhage, rather than to respond to environmental oxygen changes. Similarly, HPV, in which the HIF system plays a role, is a process evolved at sea level and is fundamental for the fetal circulation in utero, but it can be maladaptive in response to reductions in environmental oxygen.

Recently, Petousi et al. (68) studied ethnic Tibetans living at sea level in the UK and provided a first report of their phenotype in the absence of on-going, severe environmental hypoxia. In this study, Tibetans resident at sea level had a lower Hb concentration, a higher resting ventilation, and blunted pulmonary vascular responses to both acute (minutes) and sustained (8 h) hypoxic challenges compared with Han Chinese lowlanders. The physiological studies were complemented with cellular studies, which showed that the relative expression and the hypoxic induction of HIF-regulated genes were significantly lower in peripheral blood lymphocytes from Tibetans compared with Han Chinese. Thus this study provided evidence that Tibetans possess a hypo-responsive HIF system and demonstrated that a phenotype is present at sea level in the absence of on-going environmental hypoxia to activate the HIF system. Indeed, Tibetans are the first humans in whom a hypo-responsive HIF system has been demonstrated, and this may represent an evolutionary resetting of the HIF system to operate within a hypoxic environment.

Nonetheless, for the genes that have undergone natural selection in Tibetans, neither the functional variants nor the mechanisms by which they may be operating have been identified to date. In the case of *EPAS1*, all of the genetic variants reported in Tibetans to date are found in noncoding regions, either within introns of *EPAS1* or downstream of *EPAS1*; whole exome sequencing (96) or targeted sequencing of the full-length *EPAS1* gene (62) in Tibetans failed to reveal any coding genetic variants as functional candidates. Thus it is likely that “functional” genetic variation in Tibetans might lie in regulatory regions and actually affect transcription of the *EPAS1* gene itself, as opposed to downstream HIF-2α structure and function. In keeping with such a possibility, Petousi et al. (68) reported lower HIF-2α mRNA expression in peripheral blood lymphocytes from Tibetan than from Han Chinese volunteers, which may be related to lower levels of transcription of *EPAS1*. In the case of *EGLN1*, two coding variants have been described of particularly high frequencies in Tibetans (48, 49, 50, 94), although their effect on PHD2 protein structure and function are not yet known. Functional molecular studies are required to unravel the “causal” variants in the candidate genes and their mechanism of action. Further exploration of Tibetan physiology by integrating studies at the genomic level with functional molecular studies and whole-system phenotyping has the potential to yield important additional insights into human evolution in response to the environment of high-altitude hypoxia.

## GRANTS

N. Petousi was funded by a Wellcome Trust Clinical Training Research Fellowship (Grant 089457/Z/09/Z).

## DISCLOSURES

No conflicts of interest, financial or otherwise, are declared by the author(s).

## AUTHOR CONTRIBUTIONS

Author contributions: N.P. drafted manuscript; N.P. and P.A.R. edited and revised manuscript; N.P. and P.A.R. approved final version of manuscript.
